# Ferroportin and soluble transferrin receptor in women with first-trimester missed abortion

**DOI:** 10.17305/bb.2026.14261

**Published:** 2026-06-12

**Authors:** Ana Cuk, Lada Rumora, Ivanka Mikulic, Nikolina Penava, Ivona Cvetkovic, Vinka Mikulic, Kristina Ljubic, Ante Pusic, Andrea Prce, Dejan Tiric, Vajdana Tomic

**Affiliations:** 1 Department of Laboratory Diagnostics, University Clinical Hospital Mostar, Mostar, Bosnia and Herzegovina; 2 Faculty of Pharmacy, University of Mostar, Mostar, Bosnia and Herzegovina; 3 Department of Medical Biochemistry and Hematology, University of Zagreb Faculty of Pharmacy and Biochemistry, Zagreb, Croatia; 4 Department of Obstetrics and Gynaecology, Clinical Hospital Centre Mostar, Mostar, Bosnia and Herzegovina

**Keywords:** Iron homeostasis, ferroportin, soluble transferrin receptor, hepcidin, missed abortion, pregnancy

## Abstract

Laboratory diagnostics of iron homeostasis during pregnancy typically rely on routine parameters, which often inadequately reflect the actual maternal iron status. This study aimed to evaluate the investigative and diagnostic utility of soluble transferrin receptor (sTfR) and ferroportin (Fpn) concentrations as key indicators of iron dysregulation in women with normal early pregnancy and those experiencing missed abortion. These primary biomarkers were assessed in conjunction with standard laboratory parameters of iron status across the study groups. Notably, Fpn concentration had not previously been determined in women with either normal or abnormal pregnancies. The study enrolled 45 women with missed abortion and 45 women with normal first-trimester pregnancies. Peripheral blood concentrations of iron (Fe), unsaturated iron-binding capacity (UIBC), ferritin, hemoglobin (Hb), transferrin, and sTfR were measured using automated analyzers, while hepcidin and Fpn were quantified using ELISA kits. Transferrin saturation (TSAT), the sTfR/log ferritin index (sTfR-F index), and the ferroportin to soluble transferrin receptor ratio (Fpn/sTfR) were calculated. In the missed abortion group, Fpn (*P <* 0.001), Fe (*P ═* 0.008), TSAT (*P ═* 0.018), and Fpn/sTfR (*P <* 0.001) were significantly decreased, whereas sTfR (*P ═* 0.001) and the sTfR-F index (*P ═* 0.003) were significantly increased compared to women with normal pregnancies. Proposed multiparameter models incorporating sTfR and Fpn, or sTfR, hepcidin, and Fpn, accurately classified over 80% of cases and demonstrated excellent area under the curve (AUC) values of approximately 0.9. These findings indicate that Fpn and sTfR may serve as valuable investigative biomarkers for identifying dysregulation of iron homeostasis in early pregnancy.

## Introduction

A significant number of pregnancies conclude in unexplained spontaneous abortion. The majority of miscarriages occur in early pregnancy, with an 11%–22% risk up to the 20th week of gestation, peaking before the 14th week [1, 2]. Missed abortion is a unique form of spontaneous abortion, characterized by the asymptomatic death of the embryo or fetus (absence of cardiac activity) without sufficient uterine contractions for expulsion, distinguishing it from other types that involve heavy bleeding [3, 4].

During a normal pregnancy, maternal iron homeostasis undergoes substantial physiological adaptations to support fetal development. In healthy pregnancies, serum iron (Fe), ferritin, and transferrin saturation (TSAT) typically decrease due to plasma volume expansion and increased fetal demand. Current laboratory diagnostics for iron homeostasis in pregnancy rely on measuring Fe, hemoglobin (Hb), and ferritin concentrations. However, these parameters alone are insufficient for a comprehensive assessment of maternal iron status, which critically influences embryonic organogenesis and fetal growth, particularly in early pregnancy and throughout its later stages [5].

Maternal iron deficiency during pregnancy poses a risk for insufficient iron supply to the embryo, given that placental iron transfer is crucial for early embryonic development [6].

Iron absorption and tissue distribution are primarily regulated by the interaction of hepcidin and ferroportin (Fpn). Hepcidin, a liver hormone, plays a central role in iron homeostasis. Iron and hepcidin form a classical endocrine feedback loop: iron excess increases hepcidin levels, which inhibits further iron absorption and release from stores. Conversely, iron deficiency leads to lower hepcidin concentrations, thereby increasing plasma iron availability. Hepcidin has also been shown to be important in regulating placental iron transport [6–10]. Recent research further suggests a potential link between dysregulation of iron homeostasis regulators, particularly hepcidin, and early spontaneous abortion [11, 12].

The hepcidin receptor is Fpn, which is also the sole known cellular iron exporter. Recent studies have elucidated the structural and functional insights of hepcidin-bound Fpn [13]. Fpn has been identified and expressed in iron-storing and transporting tissues, including the placenta [14, 15]. Its localization on the basal/fetal side of the syncytiotrophoblast underscores its critical role in transplacental iron transfer [16–19].

Iron homeostasis is a dynamic regulatory process involving transferrin to transport iron between sites of absorption, recycling, and storage to sites of utilization. Transferrin is the primary iron transporter in plasma [19]. Iron from the maternal circulation, bound to transferrin, is taken up by transferrin receptor 1 (TfR1), located on the apical/maternal side of the syncytiotrophoblast. Following endocytosis, iron is released from transferrin and transported to the cytoplasm. Within the cytoplasm, iron can be stored in ferritin, associated with heme, incorporated into the labile iron pool, or transported to the fetal circulation via Fpn. Simultaneously, TfR1 and transferrin are recycled back to the apical membrane of the syncytiotrophoblast, with transferrin subsequently released into the maternal circulation [20].

Soluble transferrin receptor (sTfR) is a truncated monomer of its tissue counterpart, lacking the first 100 amino acids. Serum sTfR concentrations have been demonstrated to be proportional to cellular/tissue TfR1 levels [21, 22]. sTfR concentrations remain stable until iron stores are depleted. While ferritin and TSAT are primary markers for diagnosing iron deficiency, sTfR offers valuable supplementary information about iron status. Although not universally available and requiring careful interpretation, an increase in sTfR can precede a significant drop in Hb levels. Therefore, unlike serum ferritin, which provides limited information on iron stores, sTfR can accurately assess iron status.

Currently, it is unclear whether abnormal changes in iron metabolism contribute to adverse pregnancy outcomes or if, conversely, abnormal pregnancy causes dysregulation in iron-related parameters. Thus, future studies are needed to clarify this causal relationship. Sangkhae et al. [6, 23] proposed a possible mechanism of transplacental iron transport in mouse and human placenta models, emphasizing the importance of key iron transporters: TfR1 for iron import and Fpn for iron export. The authors found that during iron deficiency, the placenta downregulates Fpn and upregulates TfR1 to maintain its own iron homeostasis, leading to reduced iron availability for fetal transfer. This placental response, which prioritizes placental iron retention despite fetal iron deficiency, is of critical importance, leading to the designation of the placenta as “selfish” [24].

Our hypothesis posits that decreased serum concentrations of Fpn and increased serum concentrations of sTfR serve as early indicators of iron homeostasis disorders and could be crucial for understanding early missed abortion. The primary aim of this study was to evaluate the investigative and diagnostic potential of sTfR and Fpn concentrations—as key molecular components regulating trans-placental iron transfer and central indicators of iron dysregulation—in women with normal early pregnancies and those experiencing missed abortion. Our secondary aim was to propose a model incorporating various iron-related parameters to assess the combined diagnostic potential of these markers in first-trimester missed spontaneous abortion.

## Materials and methods

### Study population

Participants were categorized into a normal pregnancy (control) group and a missed spontaneous abortion group. The sample size was estimated using MedCalc software. To align with the research objective and planned statistical analyses, a pilot study was conducted to determine the standard deviation of the differences in mean Fpn between the two groups. Strict criteria for sample size calculation included an assumed α ═ 0.05 and a research power of 90%.

For the missed abortion group, blood samples were collected at the Clinic for Gynecology and Obstetrics following ultrasound confirmation of an early-retained spontaneous abortion. For the control group, blood samples were collected at the Transfusion Clinic Centre of the University Clinical Hospital (UCH) Mostar (Bosnia and Herzegovina). Samples for both groups were collected in the morning. In the missed abortion group, blood was drawn after ultrasound confirmation of missed abortion and prior to the induction of abortion with mifepristone or misoprostol. All control group subjects were contacted two months after consenting to participate to confirm a normal course of pregnancy and the absence of subsequent first-trimester spontaneous abortion. Inclusion criteria for both groups were age 18–45 years, gestational week < 14, singleton pregnancy, and Hb > 11 g/dL. Additional inclusion criteria for the control group included normal pregnancy with ultrasound-confirmed normal embryonic or fetal development and a history of previous normal pregnancies without spontaneous abortions. For the missed abortion group, inclusion criteria were retained spontaneous abortion with ultrasound-confirmed termination of embryonic or fetal development (absence of cardiac activity) and induction of abortion with mifepristone or misoprostol. Exclusion criteria for both groups encompassed bleeding, anemia, inflammation, hypertension, diabetes, ultrasound-visible fetal anomalies, thyroid dysfunction, urinary infection, uterine malformations, malignant diseases, acquired and hereditary thrombophilia, autoimmune diseases, artificial insemination, and iron supplementation, as well as CRP > 5 mg/L. Based on these criteria, participants had no relevant pregnancy history.

### Methods for determination of iron status parameters

We determined the concentrations of Fe, unsaturated iron-binding capacity (UIBC), ferritin, and Hb, which are routinely used in clinical practice for assessing iron in pregnancy. Additionally, transferrin and sTfR, also used in clinical practice but not typically in pregnancy, were measured, along with hepcidin and Fpn concentrations, which are not routinely used clinically. We also calculated total iron-binding capacity (TIBC), TSAT, soluble transferrin receptor/log ferritin index (sTfR-F index), and the ferroportin to soluble transferrin receptor (Fpn/sTfR) ratio. The following parameters were calculated: TIBC = Fe (µmol/L) + UIBC (µmol/L); TSAT (%) ═ [Fe (µmol/L)/TIBC (µmol/L)] × 100; sTfR-F index = sTfR concentration / log ferritin concentration. The Fpn/sTfR ratio was calculated directly from measured Fpn and sTfR concentrations without harmonization of measurement units. This ratio was used as an exploratory research-derived parameter for comparative analyses between groups, not as a validated clinical biomarker. Most parameters were determined at the Department of Laboratory Diagnostics, UCH Mostar, while Fpn and hepcidin were measured at the University of Zagreb Faculty of Pharmacy and Biochemistry. Hb concentration was determined from whole blood with K3-ethylenediaminetetraacetic acid (EDTA) on a Sysmex XN 1000 hematology counter (Wakinohama-Kaigandori Chuo-ku, Kobe 651–0073, Japan) on the day of sample collection. Serum samples for biochemical markers were obtained by centrifugation of whole blood without anticoagulant for 10 min at 3500 rpm, then stored at –80 ^∘^C until analysis (performed within 6 months). Serum concentrations of Fe, UIBC (photometric method), and CRP (CRP Latex reagent kit) were determined using a Beckman Coulter DxC 700 AU automatic analyzer (Beckman Coulter, Brea, USA). Transferrin concentration was determined by the immunoturbidimetric method on an Alinity ci automatic analyzer (Abbott, Germany). Serum sTfR concentration was determined nephelometrically on a BN II System automatic analyzer (Siemens, Erlangen, Germany), and ferritin concentration using the chemiluminescence method on a Vitros 3600 analyzer (Ortho-Clinical Diagnostics, Buckinghamshire, UK). Concentrations of Fpn and hepcidin in serum were determined using the enzyme-linked immunosorbent assay (ELISA) sandwich method (comprehensive technical details and protocol for Fpn and hepcidin measurement are provided in the Supplemental data).

### Ethical statement

The study received ethical approval from the Ethics Committee of UCH Mostar and the Ethics Committee for Experimentation of the University of Zagreb Faculty of Pharmacy and Biochemistry (Zagreb, Croatia), under protocol numbers 1036/21 and 251-62-03-22-19, respectively. All methodologies adhered to relevant guidelines and regulations, including the Declarations of Helsinki. Informed consent was obtained from each participant after verbal explanation and review of an information letter.

### Statistical analysis

Continuous variables were summarized as mean ± standard deviation (SD) or median with interquartile range (IQR), contingent on distribution characteristics. Given the moderate sample size, formal normality testing was interpreted cautiously and not used as the sole criterion for statistical inference. Group comparisons were conducted using Student’s *t*-test and the Mann–Whitney *U* test. Effect sizes, accompanied by 95% confidence intervals (CIs), were reported to highlight the magnitude and precision of observed differences. To address multiple testing, *P*-values were adjusted using the Benjamini–Hochberg false discovery rate (FDR) procedure, and corresponding *q*-values were reported.

Univariate and multivariate logistic regression analyses were performed. All continuous variables were standardized to z-scores, and Odds Ratios (OR) with their 95% CIs are reported per one SD increment for both univariate and multivariable logistic regression analyses. To mitigate optimism and control for overfitting, internal validation was systematically performed for all developed multiparameter models using non-parametric bootstrap resampling with 1000 replications. Bias-corrected and accelerated 95% CIs were calculated for all Area Under the Receiver Operating Characteristic Curve (AUC) values. For each model, the optimal classification cut-off point was determined using the Youden index, and overall classification accuracy, sensitivity, and specificity were calculated based on these bootstrap-validated optimal thresholds.

In multivariate logistic regression, the analysis was adjusted for maternal age (maternal age-adjusted multivariate logistic regression). This involved performing analyses both with and without the maternal age parameter to assess the independent effect of other covariates on the outcome. Model 1 included only routine biochemical parameters of iron status that demonstrated significance in the univariate analysis. Model 2 encompassed parameters that reached significance in the univariate analysis, irrespective of their standard use in laboratory practice. To avoid omitting variables that might be non-significant individually but crucial within a multivariate logistic regression [25], Model 3 combined all examined parameters regardless of their univariate significance. Additionally, a sensitivity analysis was performed using multivariable logistic regression to evaluate the independent prognostic value of the Fpn/sTfR ratio relative to its individual components. All differences with a *P*-value (or *q*-value) < 0.05 were considered statistically significant. Data analysis was performed using MedCalc^®^ Statistical Software version 22.006 (MedCalc Software Ltd, Ostend, Belgium).

## Results

This research revealed statistically significant differences in the concentrations of sTfR and Fpn between the examined groups. The median serum sTfR concentration in women with a normal pregnancy was 0.84 (0.62–1.08) mg/L, compared to 1.02 (0.86–1.20) mg/L in women experiencing missed abortion (*P ═* 0.001, *q* ═ 0.012) (Figure 1). Conversely, the median serum Fpn concentration was 11.05 (7.42–21.35) µg/L in normal pregnancies, significantly higher than the 6.66 (4.44–8.99) µg/L observed in women with missed abortion (*P <* 0.001, *q* ═ 0.001) (Figure 1).

**Figure 1.**
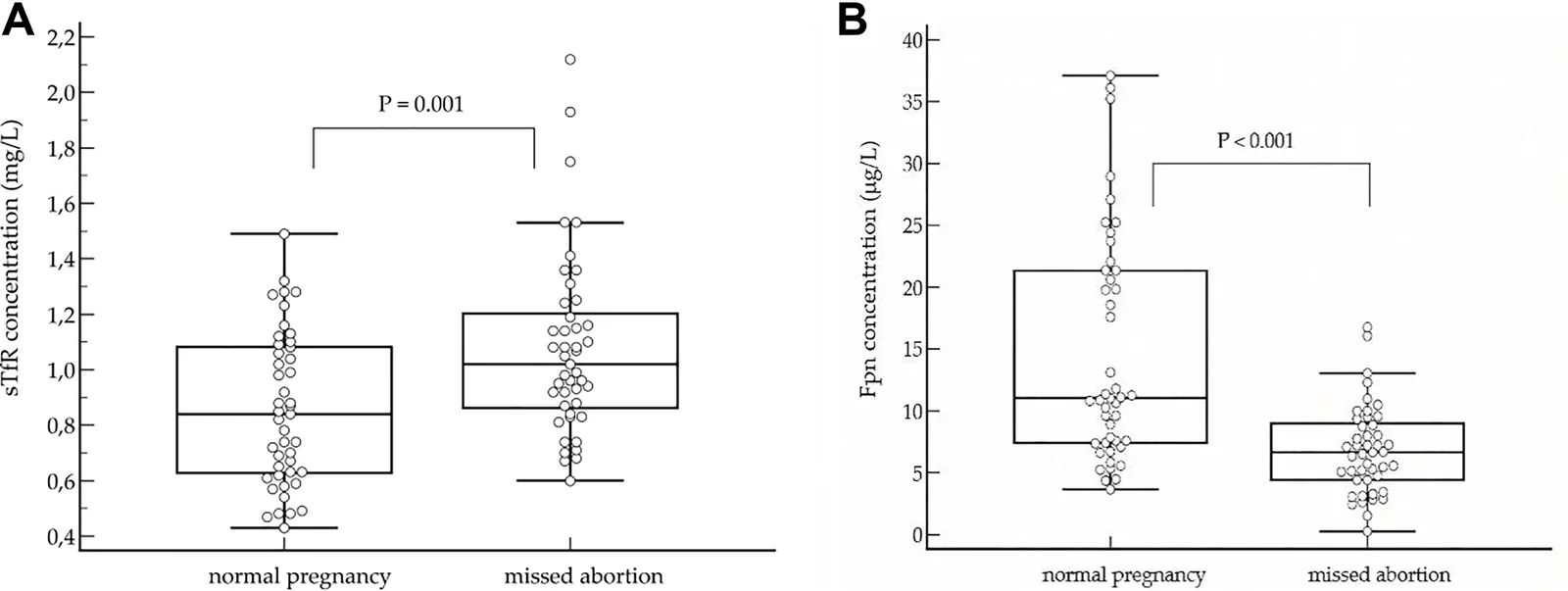
**Missed abortion is associated with increased serum sTfR and decreased serum Fpn concentrations.** Serum concentrations of (A) sTfR and (B) Fpn in women with normal first-trimester pregnancy and women with missed abortion before 14 gestational weeks (*n* ═ 45 per group). Groups were compared using the Mann–Whitney *U* test. Median sTfR concentrations were 0.84 (0.62–1.08) mg/L in normal pregnancy and 1.02 (0.86–1.20) mg/L in missed abortion (*P ═* 0.001; *q* ═ 0.012). Median Fpn concentrations were 11.05 (7.42–21.35) µg/L in normal pregnancy and 6.66 (4.44–8.99) µg/L in missed abortion (*P <* 0.001; *q* ═ 0.001). *q* values were calculated using the Benjamini–Hochberg false discovery rate procedure. **Abbreviations:** Fpn, ferroportin; IQR, interquartile range; sTfR, soluble transferrin receptor.

Further comparison between the control and study groups demonstrated statistically significant differences in serum concentrations of Fe, TSAT, sTfR-F index, and Fpn/sTfR. No other parameters exhibited statistically significant differences (Table 1).

**Table 1:** Biochemical parameters in women with normal pregnancy and missed abortion

**Parameter**	**Normal pregnancy** ***n* ═ 45**	**Missed abortion** ***n* ═ 45**	**Difference *(95 % CI)***	***P*-value**	*q*-value*
Fe (µmol/L)	17.6 (13.2–24.0)	12.2 (8.5–19.3)	−4.30 (−7.60 to −1.30)	**0.008**	**0.024**
UIBC (µmol/L)	45.1 ± 11.7	49.4 ± 12.8	4.26 (−0.77 to 9.29)	0.095	0.162
TIBC (µmol/L)	63.8 ± 8.9	63.8 ± 8.8	−0.02 (−3.75 to 3.69)	0.987	0.987
ferritin (µg/L)	18.6 (11.3–29.3)	15.1 (10.3–22.6)	−3.40 (−8.30 to 0.70)	0.103	0.154
Hb (g/dL)	12.7 ± 0.7	12.5 ± 1.0	−2.66 (−6.43 to 1.14)	0.169	0.225
transferrin (mg/dL)	285.4 ± 46.7	293.3 ± 47.7	7.86 (−12.78 to 28.51)	0.446	0.486
TSAT (%)	26 (19–38)	19 (12–33)	−6.65 (−11.66 to −1.28)	**0.018**	**0.043**
sTfR-F index	0.69 (0.44–0.83)	0.82 (0.67–1.14)	0.20 (0.07 to 0.34)	**0.003**	**0.012**
hepcidin (µg/L)	3.86 (2.04–5.35)	4.69 (2.56–7.32)	0.91 (−0.33 to 2.26)	0.180	0.216
Fpn/sTfR	15.31 (10.14–22.70)	6.88 (4.09–8.50)	−8.81 (−11.82 to −6.10)	**< 0.001**	**0.001**

Data are presented as mean ± SD and IQR. Group differences are reported as effect size estimates with 95% CI. Comparisons between groups were performed using Student’s *t*-test or Mann–Whitney *U* test, as appropriate. **Abbreviations:** CI, confidence interval; Fe, iron; Fpn, ferroportin; Fpn/sTfR, ferroportin to soluble transferrin receptor ratio; Hb, hemoglobin; IQR, interquartile range; q-value, false discovery rate-adjusted *P*-value; SD, standard deviation; sTfR, soluble transferrin receptor; sTfR-F index, soluble transferrin receptor/log ferritin index; TIBC, total iron-binding capacity; TSAT, transferrin saturation; UIBC, unsaturated iron-binding capacity. *False Discovery Rate (FDR) adjusted q-values were calculated for multiple comparisons using the Benjamini–Hochberg procedure.

A statistically significant age difference was observed between the study groups. The median age for the control group was 29 (range: 25–41) years, while the missed abortion group had a median age of 35 (range: 20–43) years (*P <* 0.001, *q* ═ 0.001). Additionally, a statistically significant difference in Body Mass Index (BMI) was found (control group: 23.85 ± 1.93; study group: 22.81 ± 2.04; *P ═* 0.029). However, after applying FDR analysis to all variables, BMI no longer showed a statistically significant difference between groups (*q* ═ 0.058). No statistically significant difference in gestational week was observed between the groups; the median and interquartile range for the control group was 10 (7–12) weeks, and for the study group was 10 (9–11) weeks (*P ═* 0.180, *q* ═ 0.216).

Univariate logistic regression analysis was conducted to assess the predictive value of the investigated biochemical parameters (Table 2). sTfR and sTfR-F index emerged as the strongest predictors of missed abortion risk. Conversely, Fe, ferritin, TSAT, and particularly Fpn and Fpn/sTfR, demonstrated a protective effect.

**Table 2:** Univariate logistic regression analysis of parameters of iron homeostasis

**Parameter**	OR*	95 % CI*	***P*-value**
Fe (µmol/L)	0.97	0.95–0.99	**0.011**
UIBC (µmol/L)	1.42	0.92–2.19	0.106
TIBC (µmol/L)	0.99	0.65–1.50	0.987
ferritin (µg/L)	0.58	0.36–0.93	**0.025**
Hb (g/dL)	0.73	0.47–1.13	0.171
transferrin (mg/dL)	1.18	0.77–1.80	0.426
TSAT (%)	0.59	0.38–0.93	**0.023**
sTfR (mg/L)	2.32	1.36–3.97	**0.001**
sTfR-F index	2.93	1.40–6.12	**0.004**
hepcidin (µg/L)	1.34	0.85–2.13	0.202
Fpn (µg/L)	0.17	0.07–0.43	**<0.001**
Fpn/sTfR	0.04	0.01–0.17	**<0.001**

**Abbreviations:** CI, confidence interval; Fe, iron; Fpn, ferroportin; Fpn/sTfR, ferroportin to soluble transferrin receptor ratio; Hb, hemoglobin; OR, odds ratio; SD, standard deviation; sTfR, soluble transferrin receptor; sTfR-F index, soluble transferrin receptor/log ferritin index; TIBC, total iron-binding capacity; TSAT, transferrin saturation; UIBC, unsaturated iron-binding capacity.* ORs and 95 % CIs are expressed per one SD increment.

The diagnostic potential of the proposed multiparameter models was evaluated using multivariate logistic regression analysis, with results summarized in Table 3. All reported statistical parameters, including AUC values and classification accuracy, represent outcomes obtained after bootstrap validation and adjustment for maternal age. Model 1 exhibited an unadjusted AUC of 0.691 with an accuracy of 71.11%, and an adjusted AUC of 0.786 with an accuracy of 75.55%. Model 2 demonstrated an unadjusted AUC of 0.851 with an accuracy of 81.11%, and an adjusted AUC of 0.880 with an accuracy of 81.11%. Model 3 achieved an unadjusted AUC of 0.888 with an accuracy of 85.56%, and an adjusted AUC of 0.923 with an accuracy of 88.89%.

**Table 3:** Multiparameter models assessed via multivariate logistic regression analysis

**Unadjusted**					Adjusted^*^		
**Model**	**Parameter**	**OR**	**95 % CI**	***P*-value**	**OR**	**95 % CI**	***P*-value**
	Fe (µmol/L)	0.93	0.86–1.00	0.059	0.93	0.86–1.01	0.103
1	ferritin (µg/L)	0.57	0.32–1.02	0.061	0.60	0.32–1.11	0.105
	TSAT (%)	3.67	0.62–21.52	0.149	3.71	0.48–28.19	0.205
		The analysis yielded an AUC 0.691 (95% CI: 0.579–0.799) and an accuracy: 71.11% (S:88.89%, Sp:53.33%)**			The analysis yielded an AUC 0.786 (95% CI: 0.683–0.867) and an accuracy: 75.55% (S:71.11%, Sp:80.00%)**		
	sTfR (mg/L)	2.90	1.39–6.05	**0.004**	2.58	1.20–5.55	**0.014**
2	Fpn (µg/L)	0.15	0.06–0.41	**< 0.001**	0.14	0.05–0.42	**< 0.001**
		The analysis yielded an AUC 0.851 (95% CI: 0.757–0.920) and an accuracy: 81.11% (S:91.11%, Sp:71.11%)**			The analysis yielded an AUC 0.880 (95% CI: 0.797–0.937) and an accuracy: 81.11% (S:95.56%, Sp:66.67%)**		
	sTfR (mg/L)	2.82	1.30–6.12	**0.008**	2.29	1.03–5.09	**0.042**
	hepcidin (µg/L)	3.45	1.31–9.07	**0.012**	4.55	1.44–14.38	**0.009**
3	Fpn (µg/L)	0.07	0.02–0.27	**< 0.001**	0.05	0.01–0.27	**< 0.001**
		The analysis yielded an AUC 0.888 (95% CI: 0.803–0.947) and an accuracy: 85.56% (S:95.56%, Sp:75.56%)			The analysis yielded an AUC 0.923 (95% CI: 0.847–0.965) and an accuracy: 88.89% (S:91.11%, Sp:86.67%)		

**Abbreviations:** AUC, area under the receiver operating characteristic curve; CI, confidence interval; Fe,iron; Fpn,ferroportin; OR, odds ratio; S, sensitivity; Sp, specificity; sTfR, soluble transferrin receptor; TSAT, transferrin saturation. ORs and 95% CIs are expressed per one SD increment. * Adjusted for maternal age. ** AUC, CI, S, Sp, and accuracy were calculated following internal validation via non-parametric bootstrap resampling with 1000 replications to correct for model optimism. The optimal cut-off value was determined using the Youden index.

A sensitivity analysis employing multivariable logistic regression confirmed that the Fpn/sTfR ratio effectively captured the joint predictive variance of its underlying components without introducing spurious associations. When modeled together, both Fpn and sTfR lost statistical significance (*P ═* 0.269 and *P ═* 0.668, respectively), whereas the Fpn/sTfR ratio remained a highly significant independent predictor (*P <* 0.001). Furthermore, this combined model yielded a nearly identical area under the receiver operating characteristic (ROC) curve (AUC: 0.852, 95% CI: 0.763–0.921; accuracy: 80.00%) compared to the model with individual components alone (AUC: 0.851, 95% CI: 0.757–0.920; accuracy: 81.11%), demonstrating that the ratio effectively maintains full discriminative capacity.

## Discussion

Recent literature underscores the critical importance of understanding iron homeostasis during pregnancy and its impact on adverse pregnancy outcomes [26]. This study assessed hepcidin concentrations in women with both normal and abnormal pregnancies. Additionally, it comprehensively evaluated various iron homeostasis parameters in women experiencing early (before the 14th gestational week) missed abortion, a specific type of spontaneous abortion characterized by the absence of vaginal bleeding.

Our results indicate that Fe and TSAT as well as Fpn and the Fpn/sTfR ratio, were significantly decreased in the missed abortion group compared to women with normal pregnancies in the first trimester. Conversely, sTfR and the sTfR-F index were significantly increased in the missed abortion group. The Fpn parameter and the Fpn/sTfR ratio should be interpreted cautiously, as they represent exploratory research variables rather than established clinical tools. Currently, only ELISA kits are available for determining Fpn concentration from serum or plasma. Standardization of the method and verification of the test according to the Clinical and Laboratory Standards Institute (CLSI) EP15-A3 protocol for medical laboratory tests are essential. Given iron’s multifaceted roles in physiological functions, understanding serum/plasma Fpn concentrations is crucial for elucidating various disorders of iron homeostasis, particularly in the context of pregnancy. While Fpn has not previously been expressed as a ratio with sTfR, we propose that the Fpn to sTfR ratio may hold significance in evaluating the intensity of iron homeostasis disruption. However, further studies with larger participant cohorts and/or designs amenable to ROC analysis, including sensitivity, specificity, and cut-off point data, are necessary to properly evaluate the Fpn/sTfR ratio’s clinical utility.

To the best of our knowledge, only two papers by the same authors have determined iron status parameters in women with spontaneous abortion, though the specific type of abortion was not specified [11, 12]. These studies enrolled Chinese non-pregnant women, pregnant women in the first trimester, women who experienced spontaneous abortion in the first trimester [11, 12], and pregnant women in the second trimester [11]. Iron levels in the first [11, 12] and second trimesters [12] were comparable to those in non-pregnant women but significantly increased in women with spontaneous abortion. Hepcidin concentration was similar [12] or elevated [11] in the first trimester and decreased in the second trimester [11] compared to non-pregnant women, yet it was increased in the spontaneous abortion group [11, 12]. Furthermore, TIBC was decreased, while TSAT and ferritin were increased in women with spontaneous abortion compared to pregnant women in the first trimester [11]. Data regarding iron supplementation were unavailable for one of these studies [11], and in the other, only a small number of women received vitamin supplementation, with no specific indication of whether iron was included, although it could be presumed [12].

Two additional studies investigated indicators of iron status during normal pregnancy in European and African cohorts [27, 28]. However, these studies primarily focused on changes across all three trimesters or the postpartum period, which limits direct comparisons with our first-trimester data. Moreover, while the European study [27] lacked information on iron supplementation, the African cohort [28] exclusively recruited women receiving iron supplements. Discrepancies in results between previous research involving women with normal pregnancy [27, 28] and/or spontaneous abortion [11, 12] and our findings may be attributed, at least in part, to variations in study group size, ethnic background (e.g., Caucasian vs. African), iron supplementation practices, presence of iron deficiency anemia, and the specific type of spontaneous abortion. Our study exclusively included women with missed abortion from an Eastern European Caucasian population, and both anemia and iron supplementation were among our exclusion criteria.

In the current research, univariate logistic regression analysis was performed to evaluate the diagnostic potential of the examined parameters. This analysis revealed increased odds of missed abortion for sTfR and sTfR-F index, while Fe, ferritin, TSAT, Fpn, and Fpn/sTfR exhibited an odds ratio less than 1, with Fpn and Fpn/sTfR demonstrating the most protective effect.

Subsequently, multivariate logistic regression analysis was conducted to propose a model for identifying the best predictive parameters for missed spontaneous abortion. In Model 1, parameters lost their predictive values and exhibited the lowest predictive performance, confirming that standard iron indicators alone are insufficient for accurately identifying missed abortion. The diagnostic potential of our proposed multiparameter models underscores the benefit of combining routine parameters with novel biomarkers such as Fpn and sTfR.

Secondly, we proposed Model 2, which exclusively included Fpn and sTfR. These markers were selected due to their statistical significance in univariate analysis and their established critical roles in placental iron transport. Notably, this study represents the first report on Fpn concentrations during pregnancy, encompassing both early normal pregnancies and spontaneous abortions. While sTfR concentrations are routinely measured in clinical settings, their application is predominantly in the diagnosis and differentiation of anemias in adults. By integrating these novel potential biomarkers into our model, we demonstrated a substantial enhancement in diagnostic potential compared to relying solely on routine laboratory practices.

To mitigate the risk of omitting variables that might lack individual significance but prove crucial within a multivariate logistic regression framework, we subsequently developed Model 3. This model incorporated all examined parameters, irrespective of their univariate significance. By integrating emerging biomarkers such as Fpn and hepcidin with conventional iron indicators, Model 3 maximized its diagnostic utility, achieving an exceptional age-adjusted AUC of 0.923 and an accuracy of 88.89%.

Although Model 3 exhibited marginally superior characteristics compared to Model 2, particularly concerning the percentage of correctly classified cases and AUC values, Model 2 also demonstrated robust performance. A key distinction is that Model 2 incorporates only one parameter (Fpn) not routinely included in everyday laboratory analysis, whereas Model 3 includes two such parameters (hepcidin and Fpn). Consequently, the presented models should be considered exploratory rather than definitive clinically applicable prediction tools. Their primary objective is to identify potentially relevant biomarker associations that warrant further rigorous investigation prior to any clinical implementation. Given the pivotal roles of Fpn and sTfR in placental iron transport, this research contributes to a deeper understanding of iron homeostasis and its availability to the embryo/fetus during early pregnancy.

### Limitations

The limitations of this research include its cross-sectional study design and the moderate sample size, which was constrained by the limited availability of women presenting with this specific subtype of missed abortion.

## Conclusion

The novel findings from our study regarding the differential concentrations of these two primary transporters highlight Fpn and sTfR (which is proportional to TfR1) as potential indicators of maternal iron bioavailability. Further research with a larger cohort is imperative to comprehensively evaluate these exploratory associations before considering any potential clinical application.

## Supplemental data

The Human Fpn ELISA Kit (AssayGenie, Cat. Code HUES03226, Dublin 2, Ireland) was employed to quantify Fpn concentrations. Standards or samples were added to microplate wells and incubated with a specific antibody. Absorbance was then measured spectrophotometrically at 450 nm ± 2 nm. A calibration curve was generated through serial dilutions of working solutions, with recommended concentrations of 20, 10, 5, 2.5, 1.25, 0.63, 0.31, and 0 µg/L (the latter serving as a blank). The assay demonstrated a sensitivity of 0.19 µg/L. Inter-assay coefficients of variation (CV) for low, mid-range, and high-level samples were 5.43%, 5.19%, and 3.14%, respectively, while intra-assay CVs were 6.12%, 4.50%, and 4.35%, respectively. Samples exceeding 20 µg/L were diluted 1:2, and their concentrations were determined from the standard curve, adjusted by the dilution factor.

Hepcidin concentrations were measured using the Hepcidin 25 (bioactive) HS ELISA Kit (DRG, Ref. EIA-5782, Germany). Absorbance was measured spectrophotometrically at 450 nm ± 10 nm. The calibration curve was established using five standard concentrations: 0, 1, 3, 9, 27, and 81 µg/L. The assay range was 0.153–81 µg/L. Analytical sensitivity, determined by subtracting two standard deviations from the mean of 20 replicate analyses of the zero standard, was 0.153 µg/L. Inter-assay CVs for four samples were 14.4%, 10.5%, 9.5%, and 11.2%, respectively, and intra-assay CVs were 3.8%, 4.1%, 2.2%, and 0.8%, respectively.

Only residual serum aliquots from routine diagnostic tests were utilized in this study. All serum parameters were measured within six months of collection.
